# Most common inappropriate drug usage factors in anthelmintic treatment on sheep farms in Latvia

**DOI:** 10.14202/vetworld.2022.244-251

**Published:** 2022-02-04

**Authors:** Līga Kovaļčuka, Dace Keidāne, Alīna Kļaviņa, Marta Barbara Grasberga, Armands Vekšins

**Affiliations:** 1 Clinical Institute, Faculty of Veterinary Medicine, Latvia University of Life Sciences and Technologies, K. Helmana street 8, Jelgava, LV-3004, Latvia; 2 Institute of Food and Environmental Hygiene, Faculty of Veterinary Medicine, Latvia University of Life Sciences and Technologies, K. Helmana street 8, Jelgava, LV-3004, Latvia

**Keywords:** anthelmintics, drug resistance, drug usage errors, sheep

## Abstract

**Background and Aim:**

There is little understanding about antiparasitic drug prescription trends and implementation to reduce possible drug overuse or misuse worldwide. This study aimed to review sheep parasite control strategies and antiparasitic drug use habits in Latvia. To the best of the author’s knowledge, this is the first study in the world that describes how antiparasitic drugs are used and what are the most common drug usage errors in a sheep farm.

**Materials and Methods:**

A semi-structured questionnaire was designed to collect relevant information from face-to-face interviews to assess 22 sheep farmers’ knowledge and management procedures in farms. We collected information about animal feeding, herding, parasite diagnostics, and antiparasitic drug usage. The questionnaire summary included information on pasture use, parasite control management, and anthelmintic drug choice/use.

**Results:**

Only 36% of farms regularly managed parasite control by analyzing fecal samples for parasites, but prophylactic dewormingwas employed in all farms. Ivermectin, albendazole, levamisole, and monepantel were used on the farms and most of the farms were multidrug users; 77.3% of the farms used albendazole and 72.7% used ivermectin.

**Conclusion:**

The results indicated a lack of parasitological examination and parasite control of the flock, mostly empiric drug selection, incorrect dosing, inaccurate drug administration, drug storage, and use errors. A proactive approach to herd health planning, regular parasitic control, and prophylactic measures may benefit farmers and veterinarians.

## Introduction

Parasitic infections are a widespread concern in veterinary medicine, including sheep medicine [1]. Endoparasites affect animals and influence overall health, production, and animal welfare [2,3]. Gastrointestinal parasites are one of the major challenges in sheep-health management because of the prevalence of anthelmintic resistance in many parts of the world, especially in European countries [4-8], Northern Europe [9,10], and Baltic states (i.e., Lithuania and Estonia) [11,12]. Unfortunately, drug tolerance increases not only to a single drug but also often to two or more classes of broad-spectrum anthelmintics [3,13-16], therefore decreasing animal productivity and inducing an economic loss in livestock production systems [17].

The golden age of veterinary antiparasitic drug discovery is drawing to an end; therefore, prescription-only veterinary drug use in Latvia and Europe is an area of increasing focus for the veterinary profession, agricultural sector, government, and food retailers. The agricultural sector is a significant user of antiparasitic drugs. According to the latest information in the Latvian Food and Veterinary Service (FVS) [18] drug register, there are 658 different prescription-only veterinary antiparasitic drugs, of which only 21 anti-helminthic drugs are licensed for use in sheep, comprising five active ingredients: ivermectin, levamisole, albendazole, closantel, and monepantel [18]. All the licensed drugs are broad-spectrum. Ivermectin, for example, was introduced as a veterinary drug in 1981 to target endo- and ectoparasites and was found to be highly effective against the most prominent gastrointestinal nematodes in sheep and goats [19,20]. Albendazole is an older generation antiparasitic drug used for respiratory and gastrointestinal nematodes. In many parts of the world, the salicylanilide formulation, closantel, is an important anthelmintic that is extensively used to control *Haemonchus* sp. and *Oestrus ovis*, infestation in ruminants [21]. The amino-acetonitrile derivative, monepantel, is a relatively new broad-spectrum anthelmintic drug that targets gastrointestinal nematodes in sheep and goats, including adults and L4 larvae of the most prevalent species. The key feature of monepantel is its full effectiveness against strains of nematodes resistant to traditional benzimidazoles, levamisole, macrocyclic lactones, and closantel [22].

While antiparasitic drug resistance is recognized as a global threat, little has been done to improve the understanding of antiparasitic drug prescription trends and implementation, with an overall goal to reduce possible drug overuse or misuse. Several possible factors influence anthelmintic drug resistance, including incomplete qualitative clinical animal examinations and fecal parasite examination. In addition, factors concerning antiparasitic drug use are important: Correct antiparasitic agent choice, intensive broad-spectrum drug usage, drug over-or underdosing, drug-drug, and drug-feed interactions, etc. Importantly, using incorrect routes of administration have led to the development of selective resistant parasites and this has become a growing medical and economic problem on sheep and goat farms in many countries [12,23-25]. Anthelmintic post-treatment actions, such as field herd management, should also be addressed [26,27].

Therefore, this study aimed to investigate the weak points of the anthelmintic drug treatment process, emphasizing the errors of inappropriate drug usage.

## Materials and Methods

### Ethical approval and Informed consent

Ethical approval was not required to conduct this study. In all cases, written consent was obtained from the farm owners for the study.

### Study period and location

This study was conducted in September and October of 2019 and 2020, in Latvia, including all historical and cultural regions (Kurzeme, Zemgale, Vidzeme, and Latgale). Zemgale is a typical crop-growing area with several sheep farms.

### Selection of farms

**Figure-1:**
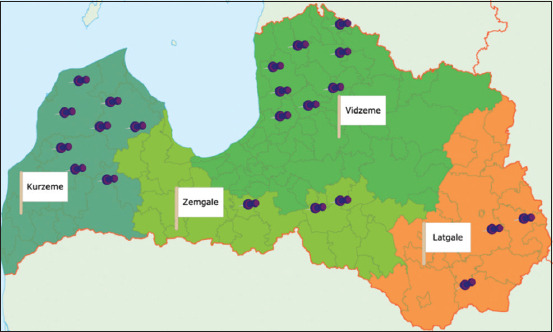
Selected study sheep farm distribution in the territory of Latvia [Source: https://lv.wikipedia.org/wiki/Attēls:Latvijas_regioni_karte.png].

A total of 28 sheep farms were randomly selected by the Agriculture Data Center of Latvia. Before the visit, six farms dropped out of the study because of the owner’s preference. Data for the remaining 22 farms were included in the analysis (Vidzeme – 8 farms; Kurzeme – 8 farms; Zemgale – 3 farms; and Latgale – 3 farms). The distribution of farms is summarized in Figure-1.

The selection of farms was based on purposive sampling based on varying herd sizes, production levels, and management practices (14 biological and eight conventional). Different sized farms were selected, including nine small (0-100 sheep), ten medium (100-300 sheep), and three large (more than 300 sheep) farms.

### Preparation of the questionnaire

A semi-structured questionnaire with multiple-choice and open-ended questions was designed to collect relevant information from in-person interviews and evaluate farmers’ knowledge and management procedures related to animal feeding, herding, parasite diagnostics, and antiparasitic drug usage.

### Data collection

On the day of the visit, a structured face-to-face interview was conducted with the self-identified main treatment decision-maker (hereafter called farmers, owner, and responsible people), to gather information on farm demographics, management practices, animal health, overall productivity, and drug use. The questionnaire included information on the farm (size, direction of activity, sheep breed, etc.), animal feeding and welfare, parasite control, drug procurement, storage, and use. In Latvia, we do not use vaccination against parasites; from other vaccines, tetanus is voluntary and was not included in the questionnaire.

The interview was conducted and performed by the same person-project manager/veterinarian. Data were collected during the 2019-2020 autumn period (September and October). Meteorological data are shown in Table-1.

**Table 1:** The average air temperature, precipitation, and relative humidity in Latvia in September and October of 2019 and 2020 [Source: https://videscentrs.lvgmc.lv].

	2019			2020		
	
	September	October	Year	September	October	Year
Average air temperature t°C	14.4	9.8	8.2	12.4	8.5	8.8
Average precipitation, mm	49.8	72.7		82.1	81.4	
Average relative humidity, %	82	87		79	87	

### Statistical analysis

For descriptive statistics, data analysis was performed using Microsoft Excel software version 2016 (Microsoft Office, USA) and Statistical Package for the Social Sciences software version 20.0 (IBM Corp., NY, USA). Responses to the questionnaire were presented as simple frequencies. Data were checked for normality. Calculations were performed using Excel. Given the cross-sectional, point-prevalence nature of the data set and the fact that the study farms were not intended to be a representative sample of the population, the presented calculations were descriptive and no inferences on causality were made.

## Results

Data were collected and analyzed to assess the farmers’ perspective on antiparasitic drug usage and factors related to animal feeding and herding. All 22 farms that were included in this study were engaged in meat production, but for 15 of the farms (68.18%), meat production was the only farm activity. Four farms (18.1%) were involved in wool production in parallel with meat production. Three farms (13.63%) in Vidzeme were also involved in animal breeding.

Across all Latvian sheep farms, the most popular breed was the Latvian dark-head sheep (81.81%) and the second most commonly held breeds were Texel or Merino (36.4%). Rarer-held breeds were Oxfords (13.63%), Romanovs (13.63%), Il-de-France (13.63%), and less than 10% were Suffolk, Borseti, German blackhead, and Estonian dark-head sheep. Borseti and German blackhead sheep were held in only one sheep farm within the Kurzeme region.

### Sheep pasture

In all farms, responsible persons admitted that sheep were primarily grazing, and 95% of respondents used the rotational grazing pasture system. On most farms, sheep return to pasture during one grazing season. Some farm pastures were located near a pond or other source of water (59%) and 55% of farms admitted that the pasture was highly humid. Water supply in bowls was provided on eight farms (36%) where a natural source of water was inaccessible. Most respondents (82%) admitted that the intrusion of wildlife animals was rare. In four farms, sheep grazed in a pasture with other animals (cattle, horses, goats, and chickens). Livestock guard dogs were used in nine (41%) farms and the respondents confirmed that all dogs were regularly dewormed.

### Parasite control management

Only 36% of the farms managed parasitic control by regularly analyzing group fecal samples (10% from the flock). All farmers agreed that they use prophylactic deworming. On 50% of the farms, antiparasitic treatment was administered twice per year and in 27.3% just once, but in 22.7% of farms it ranged between 1 and 4 times a year; one farm in Latgale dewormed sheep 3 times/year, but two farms in Vidzeme and two farms in Kurzeme administered anthelmintic drugs 4 times/year. Of all the farms, 21.8% used feces sample examination.

Animal deworming on all farms was used as treatment and prophylaxis. Only one farm started deworming as a preventative when animals presented with clinical disease and laboratory tests confirmed parasitic infection.

Farmers used various deworming methods. In 86% of farms, deworming was carried out in a sheep shed, 9% used pasture close to the sheep shed, and some farms dewormed sheep in a special pasture. After the deworming process, 41% of farmers held sheep at the deworming place, but 10% let sheep into the same pasture where animals grazed before the procedure. After completing the parasitic control management process, one farm in Vidzeme immediately let sheep into a new pasture. However, another farm in Vidzeme held animals in a shed for a couple of days and then allowed them to access new pastures. One farm in Kurzeme specified that the animals were held in a shed after deworming and then let into the same pasture, as above. In 59% of the farms, the deworming process was done at the same time for all animals, but in 41% of the farms deworming was performed at different times for separate groups.

### Antiparasitic drug choice, storage, and dose determination

Antiparasitic drugs were chosen by the veterinarian in 70% of the farms. Respondents indicated that drugs were chosen in 65% of farms without a previous coprological examination. Based on the results of the fecal examination, drugs were selected in 9.5% of farms and 19% of farmers indicated that drug usage was up to the farmer’s decision and 4.8% of farmers use pharmacy advice or internet sources to make their decisions. Antiparasitic drugs were purchased from a veterinarian (11 farms, 50%), from a veterinary pharmacy (8 farms, 36.4%), or a wholesaler (5 farms; 22.7 %). One farm pointed out that antiparasitic drugs were imported from Russia. After purchase, the drugs were stored in the farmers’ homes (59%) or refrigerators (27.4%). Two farmers (9.1%) did not specify a drug storage place and one farmer (4.5%) stored drugs on the farm. Methods for dose determination differed between the farms as well; some farmers calculated dose based on each animal (14.2%), some did group dosing (14.2%), but on other farms animals were not scaled (71.6%), and the dose was calculated by the farmers’ visual determination and previous experience. Dosing was scaled on 50% of the farms (45% had electronic scales and 5% had mechanical scales).

### Antiparasitic drug use

Our study results show that ivermectin, albendazole, levamisole, and monepantel have been frequently used. Most of the farms were multidrug users; 77.3% of the farms used albendazole and 72.7% used ivermectin. Two farmers indicated that ivermectin, albendazole, and levamisole were also used. One farm in Zemgale specified that laboratory test results confirmed resistance to ivermectin and they switched to albendazole. Oral drug administration was performed by farm owners (59%), veterinarians (18%), farm employees (9%), and in 18% of cases farm owners together with the veterinarian or an employee. In 82% of farms, drugs were administered subcutaneously, in 50% by the owner, in 11% by farm employees, in 16.6% by a veterinarian, and in 22% by the veterinarian together with the owner. In 64% of farms, drug choice was made by the veterinarian, but on other farms, drug choice was decided by the farm owner or the pharmacy manager. Drug packages were used on the same treatment day only in 45.5% of farms, 22.7% stored drugs until the subsequent use, but 31.8% of farmers used drugs repeatedly until finished.

## Discussion

To the author’s knowledge, this is the first study in the word describing antiparasitic drug use, including the most common drug usage errors, in sheep farming medicine. In this study, data derived from 22 face-to-face interviews were included.

Data showed that all farms were engaged in meat production. Sheep meat production is an important agriculture field in the European Union (EU) and correct medicine use and prophylaxis are vital in the One Health concept. According to the Latvian Sheep Breeders Association, sheep meat production has grown in Latvia. However, the European Commission reported that in EU 2020, sheep and goat meat production was reduced by 1.5% [28].

This study questionnaire included information regarding the pasture because the grazing system and pasture rotation are factors in parasite control. In our study, 95% of the respondents used a rotational grazing pasture system, but the rotation system varied from farm to farm. Rotation of livestock through different pastures reduces parasite distribution when the system is adjusted correctly. Considering that pasture downtime should be at least 1 month, some authors specify that in the 3 months of summer, there is an increased risk of parasitic infection [29]. Our study results revealed that farm deworming tactics and pasture rotation systems differ in farms and do not always meet standard guidelines. Respondents (59%) confirmed that a natural water source should be used on farm pastures. Grant *et al*. [30] suggested that farmers should avoid natural water sources to reduce the number of snails and potentially the risk of parasitic infections.

Only 36% of farms managed parasite control by analyzing fecal samples regularly for parasitological examination; however, deworming was done in 100% of cases. In contrast to antibiotic exposure, it is thought that antiparasitic resistance develops relatively slowly under field conditions and could take anywhere between 4 and 10 years [31]. An important consideration is that there is a limited choice in antiparasitic drugs, and few have been introduced in the market over recent decades. Antiparasitic drugs are used regularly in farm animals, and according to our survey, antiparasitic treatment was implemented in 100% of cases. Similarly, other studies reported that in 58%, 67%, 93%, and 99% farmers routinely administered anti-nematode treatment [32,33]. Most farmers noticed that nematodes were the most common target of the antiparasitic treatment. However, only 9.5% of farms manage parasite control by analyzing parasitology samples regularly before initiating treatment. Therefore, only one-third of all farms in our study were informed about the actual parasites in their flock of sheep.

In 27% of farms, antiparasitic treatment was done once a year, in 50% twice a year, and in 23% it was dependent on the season (3-4 times/year). Antiparasitic treatment was applied in 33% of farms as a regular prophylactic routine, in 14% when Trichostrongylidae parasite eggs were detected in a fecal examination, and 42% had more complex answers, such as prophylactic use and after animal sickness was recorded. Finally, 14% did not give a precise answer. In 52% of farms, all were treated at the same time, 19% by animal groups at different times, and in 14% only visually sick animals were treated. In similar studies, nematodes in ewes were treated on an average twice annually [32-34], lambs three to four times annually [32-34]. Notably, antiparasitic resistance can be even 4.39 times higher in flocks with a high frequency of treatment than flocks with a low frequency of treatment [35].

Sheep drugs from five different groups were used in antiparasitic treatment, namely, ivermectin, levamisole, albendazole, closantel, and monepantel, all of which are prescription-only veterinary drugs licensed in Europe. Our survey found that most of the farms were multidrug users; over 53% of the farms generally used two drugs, ivermectin and albendazole. The questionnaire results suggested that in 72.7% of cases ivermectin was used, showing more possible antiparasitic resistance risk for ivermectin, and resistance to one particular compound may be accomplished by resistance to other members of the same group [36]. Similar studies have reported macrocyclic lactones as a commonly used anthelmintic agent (47%, 56%, 70%, and 84%) [32-34]. Benzimidazoles were reported to be used against nematodes in 7%, 21% 26%, and 31% [32-34], levamisole - from 9% to 28–31% [31-33]. Contrary, Crilly *et al*. [37] reported the use of moxidectin in 71% for the periparturient treatment of ewes.

In a study of 615 sheep farms, Lima *et al*. [38] reported that farmers administered monepantel and derquantel to 32% and 28%, respectively, of ewes and rams in quarantine. Our study evaluated Monepantel but was used only on two farms; Monepantel is one of the more recent drugs marketed, but resistance to *Teladorsagia circumcincta* and *Trichostrongylus colubriformis*, following 17 successive single treatments, has already been observed [39]. In addition, use of long-acting anthelmintic formulations, such as moxidectin injection and macrocyclic lactone capsules, confers 2.85 times higher anthelmintic resistance, than those in flocks that either did not use any anthelmintic [32-40], or only used short-acting ivermectin drench [41], proposing that long-acting formulations should be reserved for specific situations, such as for the treatment of ewes carrying multiple lambs, to prevent clinical parasitism or repeated fecal egg counts [42].

In 70% of farms, antiparasitic drugs were chosen by the veterinarian, but only 11 farmers purchased drugs from the same veterinarian practice. In Latvia, the farmers can order medicine directly from a wholesaler if the registered farm has a contract with a veterinarian. Farms in regions that bordered third world countries, disclosed the possibility of importing drugs into the EU through unconventional methods, highlighting uncontrolled, and unregistered drug use in sheep. According to the Latvian FVS announcement on the use of medicines, the veterinarian is responsible; however, the results of this study suggest that the veterinarian chose anthelmintic drugs in only 70% of cases. Stubbing and Lovatt [43] suggest that the reckless use of anthelmintics results in resistance to the chemical groups being used.

According to the Food and Drug Administration, in the “Ruminant and Equine Antiparasitic Drug Use and Resistance Survey,” it was shown that in most cases 70% of small ruminant veterinarians are prescribing antiparasitic drugs for sheep treatment, according to knowledge gained in continuing education conferences and after fecal egg count/fecal tests, 38% from drug labels and only 8% from promo [35]. Furthermore, it is important to restate that the fecal egg count reduction test (FECRT), used before and after deworming, is useful for detecting anthelmintic resistance and choosing the most appropriate medicine. Drug efficiency is based on the FECRT test (74%) and determined by the resolution of a clinical sign of parasitism if present at the time of treatment (72%), conducted fecal analysis after treatment or fecal analyses (25%, respectively), only 17% trust to owner’s opinion or clinically basing that there is no parasitism after treatment, thus confirming drug efficiency (21%). Respondents showed three methods each [35]. Our survey revealed that in most cases (70%), antiparasitic drug administration was chosen by the veterinarian. However, unfortunately, without coprological examination, drugs were chosen with parasites tests only in 9.5% of cases. In 19%, drug was chosen by the owner or farmer, and in 5%, the drug was chosen after being advised by a pharmacy or through information gathered on the internet. If most veterinarians are obtaining their knowledge in continuing education and diagnosis is based on fecal testing, then knowledge of antiparasitic drugs and their use is lacking in owners; furthermore, use of antiparasitic drug combinations without justification and adequate refugia, may facilitate antiparasitic resistance to several drugs at the same time [44,45]. Therefore, drug choice without knowledge, fecal analyses, or analyses of the herd, can lead to resistance. Furthermore, only a veterinarian can decide, how to apply the correct antiparasitic drug, its form, and the dose. Antiparasitic drugs in sheep are most commonly orally administered (drenches) [29] or injected. In our study, 59% of cases featured oral drug administration by the owner, 18% by a veterinarian, and 9% by the animal keeper. The subcutaneous injection was administered in 50% of cases by the owner, 11% by the animal keeper, and 16.6% by a veterinarian (22% by veterinarian and owner together). Therefore, competent drug administration (veterinarian or veterinarian together with owner) was made in only 27% of oral cases and 38.6% of subcutaneous administration cases. The most common errors for applying oral drugs are incomplete drug administration and splitting out. In addition, there are many possible errors when administering subcutaneous injections, including injection in wool, intramuscular injection, and intradermal injection, which can lead to differences in the drug concentration reaching the worms, increasing variability, and promoting lack of effectiveness [46,47]. Therefore, it is vital that drugs are administered by a veterinarian or a skilled person, and supervised by a veterinarian. In a study on cattle, where efficiency and plasma profiles were compared after moxidectin and lately in ivermectin administration in three different routes (oral, infection, and pour-on), significant differences were found between efficiency and drug administration routes [48-51]. These studies highlight how important it is to apply drugs correctly and use the correct protocol of drug introduction. Sub-optimal dosing can potentially lead to anthelmintic resistance; however, this has not been widely formally investigated. Leathwick and Luo [26] previously implicated these factors, and for most drugs, the “dose” is an important factor when considering drug efficacy. Unfortunately, according to our survey, the dose was individually calculated on only three farms (14.2 %) and in 14.2% of farms, the dose was set by the weight of the group’s biggest sheep. In 71.6%, animals were not precisely weighed and medicine doses were calculated by the owners’ visual assessment. It is believed that without accurate animal weighing, the calculated drug dose is inaccurate. According to the guidelines, animals have to be weighed and animals should be stratified by weight before deworming [46,52]. It is recommended to use calibrated scales to prevent inaccurate dose administration. In larger groups, where the sheep’s weight is variable, it is recommended that the heaviest sheep be weighed and that the dose is given to that sheep be given to all. Still, this method carries some risk of inappropriate dosing. Ultimately, the most accurate dosing schedule is calculated after weighing each animal [46]. The guidelines suggest using scales to measure the weight of the 3-4 heaviest sheep. Suppose the weight range is significantly different within a large group. In that case, it is recommended that the group or herd be divided into two or more groups of appropriate size and the dose should be calculated for each group according to the heaviest animal in each group [46,52].

In addition, effective and successful injection is ultimately the veterinarian’s responsibility. According to our survey, 57% of owners used automatic injection syringe machines when the owner administered injections and calibration was made in 10% of cases. An uncalibrated drench gun does not deliver the correct dose; therefore, it should be calibrated as per the manufacturer’s recommendation [29]. On 50% of farms, single syringes were used, but at least in two cases, owners admitted that they were not changing needles after injecting each animal, highlighting the potential risk of secondary infection and more painful infection due to dull injection needles.

According to the instructions for drug use, antiparasitic drugs need to be stored in dry, cool, and dark place; after opening, storage is allowed for only 2 weeks. Interview results revealed that prescription drugs were kept at home in 59% of cases, in the refrigerator in 27.4% of cases, and on the farm or the veterinarian’s premises in all other cases. Inappropriate drug storage can influence drug kinetics and drugs should be maintained only until deemed inefficient. Ivermectin should be stored in the original cardboard packaging at room temperature and protected from light; for albendazole, it is not recommended to refrigerate or freeze; levamisole should be kept at room temperature and protected from light [18]. According to the official labeling, we recognize inappropriate drug storage often because none of these drugs were stored in the refrigerator. By storing them on the farm, there is a possibility for drugs to be frozen.

Drug vials were used only on the same treatment day in 45% of farms, while 23% of farms stored drugs until the next time, and 18% of farmers used drugs repeatedly until they were completed. According to the official label, ivermectin must be used within 2 weeks after opening, and albendazole within 28 days [18].

The information obtained from the results of this research is imperative to the development of antiparasitic resistance strategies in Latvian sheep flocks. More information is needed to reduce the usage of antiparasitic agents, curb the development of resistance, and safeguard the national agricultural production. Results indicate that antiparasitic drug usage is still inconsistent on farms and this can promote resistance and adversely affect animals.

## Conclusion

We noticed many drug use errors, including the following: Lack of parasitological examination and parasite control of the flock, mostly empiric drug selection (mostly on owner’s experience), incorrect dosing, inaccurate drug administration, drug storage, and use errors. Farm owners should be better educated to increase the cooperation between veterinarians and sheep farm owners. In our opinion, a more proactive approach to herd health planning, regular parasitic control and prophylactic issues may be beneficial for both farmers and veterinarians. More in-depth work should be performed to develop deworming plans specific to the herd and based on fecal examination. Efforts should be made to educate individuals to increase the owner’s responsibility of proper and sustainable use of antiparasitics, including drug product selection, management, optimizing their use, choice of drug form, dose, and the drug administration route. Fecal testing and FEC tests should be increased on the farms. The limitation of the study is that we used a small number of farms. The authors suggest a future study with a more number of farms involvement for the in-depth information.

## Authors’ Contributions

LK, DK, and AV: Conceptualization, methodology, and writing - original draft preparation. LK, DK, MBG, and AK: Questionnaire design, farm visits, interview, data curation, equally conceived the work with literature, and drafted the manuscript. LK, DK, AK, and AV: Writing - reviewing and editing. All authors have read and approved the final manuscript.
